# Planning oral health care using the sociodental approach and the index of family living conditions: a cross-sectional study in Brazilian adolescents

**DOI:** 10.1186/s13104-015-1564-3

**Published:** 2015-10-20

**Authors:** Fernanda Nunes Marques Alves, Carla Lourenço Tavares de Andrade, Mario Vianna Vettore

**Affiliations:** National School of Public Health, Oswaldo Cruz Foundation (ENSP⁄FIOCRUZ), Rua Leopoldo Bulhões 1480, Manguinhos, Rio de Janeiro, RJ CEP: 21041-210 Brazil; Unit of Dental Public Health, The School of Clinical Dentistry, University of Sheffield, 19 Claremont Crescent, Sheffield, S10 2TA UK

**Keywords:** Oral health, Needs assessment, Health planning, Primary health care

## Abstract

**Background:**

Oral health care needs assessment is frequently restricted to clinical measures. Combining normative assessment, behavioural propensity, oral health-related quality of life and information of family living conditions may provide a better comprehensive approach of adolescent’s oral health needs assessment. The aim of this study was to compare normative methods of dental caries need with the sociodental approach in 12-year-old adolescents according to family’s living conditions in a deprived community in Brazil. In addition, dental caries need assessment using the normative method and the sociodental approach was compared between adolescents living in different living conditions.

**Methods:**

A cross-sectional survey was conducted in the Manguinhos community in the city of Rio de Janeiro, Brazil. A weighted sample of 159 participants was randomly selected to represent the population of 2004 12-year-old adolescents. Socioeconomic characteristics and living conditions of the family were assessed using the Family Development Index (FDI). Oral health-related quality of life (OHRQoL) was assessed using the generic and CS-Child-OIDP, and adolescent’s propensity to adopt oral health promoting behaviours was verified through interviews. Dental caries and treatment need were assessed normatively by clinical oral examinations (DMFT Index) and adolescents were classified into two groups (non severe or severe caries). The sociodental approach included clinical measures of caries, propensity to adopt oral health promoting behaviors and OHRQoL. Families were classified based on the FDI as ‘not severe’, ‘severe’ and ‘very severe’. Measures of caries, OHRQoL and propensity outcomes were compared between FDI groups using Chi-square and Kruskal–Wallis tests. In addition, dental treatment needs using normative method and sociodental approach were compared for the whole sample and according to FDI groups.

**Results:**

Dental caries, OHRQoL and lower propensity needs were positively associated with FDI severity. The percentages of adolescents with normative dental needs from families with ‘very severe’, ‘severe’ and ‘not severe’ FDI were 59.3, 48.4 and 17.2 % (*P* < 0.05). Using the sociodental approach, the treatment needs for the three FDI groups decreased to 8.8, 13.6 and 8.6 %, respectively (*P* < 0.05).

**Conclusions:**

Using a combination of sociodental approach and the index of family living conditions was useful for defining dental care priorities in adolescents living in deprived communities and can optimise the use of resources in dental services.

## Background

Low-income children have high levels of caries and poor access to dental care [1, 2]. In order to reduce social inequalities related to access to dental care, health care systems should consider innovative approaches to organize and deliver oral health care. Adequate oral health needs assessment facilitates the more effective use of financial and human resources to benefit the oral health of the population [3].

The sociodental approach was proposed as a new method for assessing oral health needs by integrating the impact of oral health on quality of life with normative assessment and measures of propensity to adopt health-promoting behaviours of the individuals [3–5]. Normative methods are based on clinical indices and they have been traditionally used to assess dental treatment needs. However, normative assessment of oral health needs has significant variations among dentists with regards to diagnosis and treatment needs [6] and does not reflect comprehensive concepts of oral health needs because social and subjective aspects of health, such as the impact of oral health on quality of life and well-being are not considered [3–5, 7].

Impact-related need combines normative need and oral health-related quality of life (OHRQoL) and is useful to identify people in need of immediate dental care. For instance, individuals experiencing functional limitation (e.g. difficulty chewing) or social disability (e.g. avoiding social interaction) attributed to oral conditions should be prioritised. Propensity-related need is obtained by integrating normative need with OHRQoL and behavioural propensity. Health-related behaviours (e.g. oral hygiene and diet) that influence expected dental treatment outcomes are assessed [3]. People are categorized into high, moderate and poor levels of propensity-related need. Individuals with high propensity-related need are those with adequate behavioural propensity and would benefit more from treatment whereas those with moderate and poor levels of propensity-related need have greater risk of treatment failure. Thus, for them clinical treatment must be accompanied by oral health promotion activities [4, 5].

Sociodental approach in assessing needs is an individual level method that does not take into account social conditions of the individuals such as family socioeconomic status. Using information regarding family social conditions in addition to sociodental approach can contribute in the organization and delivery of oral health care. The potential benefit is to reduce social inequalities related to access and use of oral health services because it identifies those at greater social vulnerability and in greater need of dental care.

Treatment needs assessment for caries in children and adolescents have been traditionally determined by normative methods. Dental caries remains the principal oral disease of childhood and adolescence and can lead to dental pain and tooth loss, which in turn affects children and adolescents’ quality of life [1, 2].

Likewise other progressive oral conditions, dental treatment should be offered immediately irrespective if there is impact on quality of life, the level of propensity need and the socioeconomic status. Incorporating information on OHRQoL, oral health behaviours and social position would be helpful in determining what children and adolescents should be prioritised among those with dental caries.

**Fig. 1:**
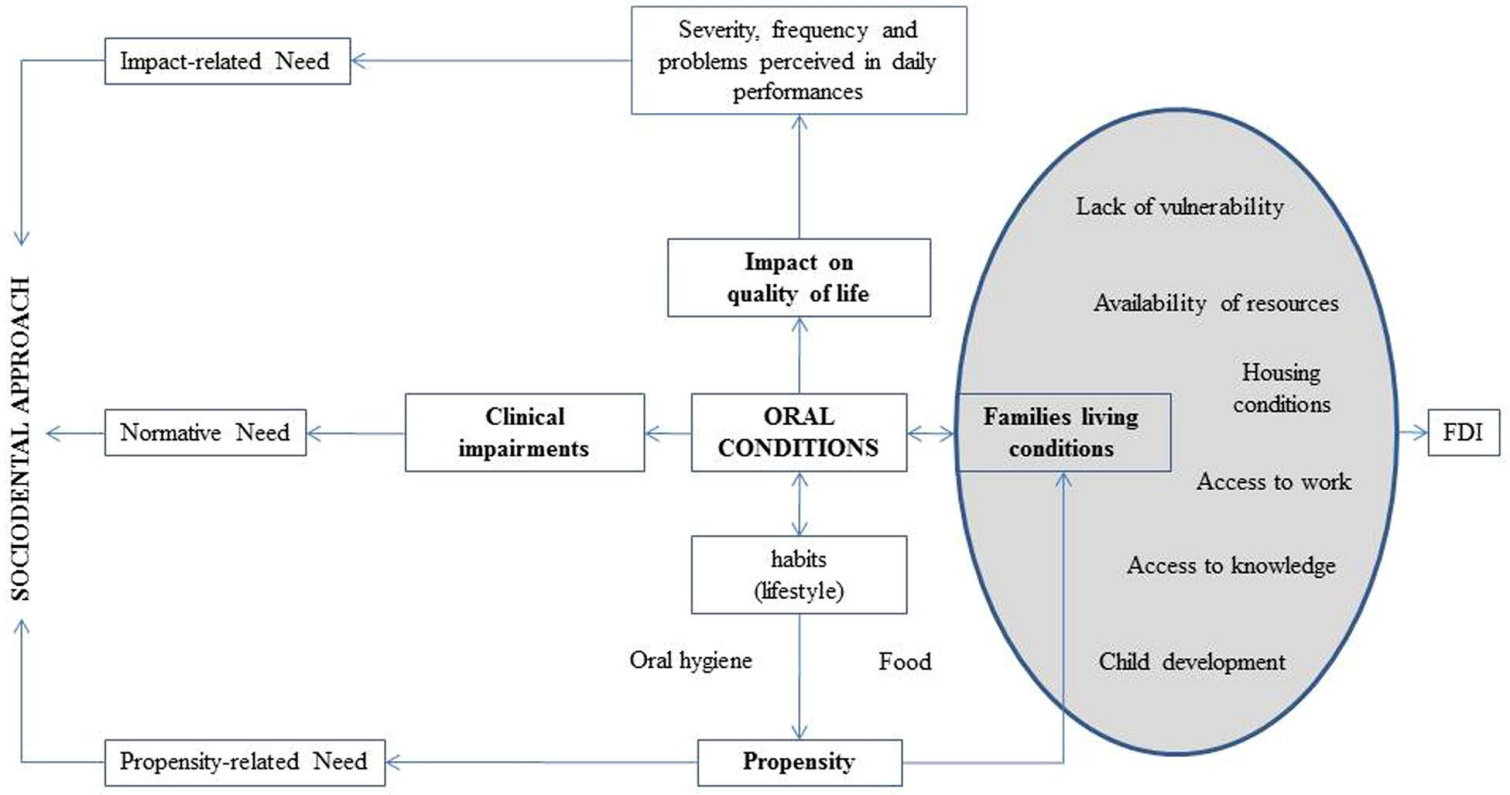
Theoretical model for oral health needs combining the sociodental approach and index of family living conditions. *FDI* family development index

The assessment of oral health needs combining sociodental approach with information of family living conditions has not been tested yet and may provide a better comprehensive approach of adolescent’s oral health needs assessment. A theoretical model of oral health needs assessment using the sociodental approach and living conditions of the family related to the organization of oral health care was developed (Fig. 1). The aforementioned framework of oral health needs incorporates the assessment of family living conditions using the family development index (FDI) [8]. The FDI is a summary score of 48 indicators combined in six dimensions of living conditions/poverty including lack of vulnerability, availability of resources, housing conditions, access to work, access to knowledge and child development [8]. The indicators use demographic and socioeconomic characteristics of the family such as presence of elderly, pregnant women or people with special care needs in the family, adults schooling, occupation, family income, children course at school [8].

The delivery of oral health care in the Brazilian health care system (Sistema Unico de Saude/SUS) is organized in primary and specialized health care units. The former, as occur in most countries, is the gateway to health care system. However, although universality, comprehensive care, equity and decentralization are the principles of SUS, health inequalities in delivering health care remains in Brazil [9].

The objective of this study was to compare normative methods of dental caries need with the sociodental approach in 12-year-old adolescents according to family’s living conditions in a deprived community in Brazil. In addition, dental caries need assessment using the normative method and the sociodental approach was compared between adolescents living in different living conditions.

## Methods

A cross-sectional survey was conducted in the Manguinhos community in Rio de Janeiro, Brazil. This community is one of the most economically deprived communities in the city of Rio de Janeiro. It is characterized by low socioeconomic status, high urban violence rates and limited access to oral health services. Manguinhos is placed 122nd in relation to the Human Development Index (HDI = 0.727) among the 126 districts in the city of Rio de Janeiro [10].

### Sample size calculation and sampling procedures

The sample size was established according to Cochran [11] using the parameters from the 2003 Brazilian Oral Health Survey [12], considering the calibrated sample weights [13]. The following parameters were set with these estimates: (1) significance level of 5 %, (2) DMFT variance of 1.8, (3) maximum error of 0.2 to estimate the DMFT mean, and (4) population size of 2004 12-year-old adolescents in the community. The sample size of 159 adolescents was estimated.

The sample was recruited in order to ensure the representativeness of the 12-year-old adolescents registered at the electronic database of Primary Health Care units in the Manguinhos community in 2009. The selection of participants was conducted through simple random sampling among adolescents registered at the Primary Health Care database. Post-hoc weight was attributed to the sample as the inverse of sampling fraction (n/N) to represent the studied population size in the community. The results are presented in expanded form for the population.

### Selection criteria

Eligible adolescents were those living in the areas covered by the Primary Health Care system of the Manguinhos community for at least 6 months. Adolescents who for any reason were unable to answer the questionnaire were excluded.

### Sociodemographic characteristics

Sociodemographic characteristics of adolescents that were recorded included sex, ethnicity, schooling, as well information of the sex of head of their families, ethnicity, adolescents’ kinship and schooling. Ethnicity classification was based on self-perception of skin colour, according to the methodology described by the Brazilian Institute of Geography and Statistics Foundation [14]. The options were ‘white’, ‘brown’, ‘black’, and ‘yellow’. Family data were: family income, housing conditions (material used in the construction of the house and access to drinking water) and financial governmental support. Family income was classified into three groups, where 1 represents the minimum wage (≤1; >1–2; >2). One Brazilian Minimal Wage corresponded to 315 US$ in 2009.

### Sociodental assessment

Sociodental approach comprises three levels of needs assessment: (1) normative need, professional judgment assessed by clinical measures; (2) impact-related need, assessed by integrating normative need with oral health-related quality of life (OHRQoL) and, (3) propensity-related need, assessed by integrating normative need with OHRQoL, the propensity for adopting oral health-related behaviours and evidence based dentistry protocols [3–5].

Oral clinical examinations were carried out by one experienced dentist (F.N.M.A.) in the households using plain dental mirror number 5, WHO millimeter dental probe (Millennium^®^) and lantern light (Heine^®^). Participants were categorized into two groups of normative treatment need for dental caries according to the severity of dental caries [15], as follows: severe caries and non-severe caries. The former group included adolescents in need of treatment for dental caries with pulp involvement (endodontic and extensive restorative treatment or dental extraction). Non-severe caries adolescents were those in need of dental restoration or white spot lesion remineralization or dental sealants.

The impact-related need assessment was performed using the validated version of child oral impacts on daily performance (Child-OIDP) for Brazilian adolescents [5, 16]. Child-OIDP was applied by the same dentist who conducted the oral examinations in two steps. Firstly, a self-applied questionnaire was used to register oral problems that affected or impaired adolescents in the last 3 months [16]. After that, face to face interview evaluated the impact of oral health on eight daily activities: eating, speaking, mouth hygiene, sleeping, maintenance of emotional state, smiling, studying and having social contact, which are grouped into physical, psychological and social dimensions [3]. Child-OIDP was designed to link specific oral problems leading to the impacts on quality of life and can be used either as a generic or condition-specific OHRQoL measure [17]. In this study, the generic and the condition-specific child-OIDP for dental caries (CS-Child-OIDP) were used. In the latter, only ‘toothache’, ‘sensitive tooth’ and ‘tooth decay’ oral problems were considered [17].

The propensity-related need regards the propensity of adolescents to adopt more oral health-related behaviours that are likely to affect dental treatment outcomes [5]. Individual interviews were used to collect data on frequency of sugar intake, frequency of tooth brushing, regular use of fluoridated toothpaste and pattern of dental attendance [5]. Those with 0–3 times daily frequency of intake of food/drink with sugar, two or more daily tooth brushing, regular use of fluoride toothpaste were classified as high propensity. The individuals classified as average propensity were those who answered at least one item at the moderate level and none at poor level. If at least one item answered was poor level, the adolescent was classified as low propensity [5].

### Family development index

Living conditions of the families were assessed through interviews with the head of family using the FDI, which is based on the human development index (HDI) [8]. In this study, FDI has been modified and adapted, considering previous studies [8, 18, 19].

The FDI score was the unweighted average of the 48 indicators varying between zero (worst situation) and one (best situation). Participants were classified in three groups based on the following cutoff points: FDI very severe (extreme poverty line, score 0 to 0.50), FDI severe (poverty line, score from 0.51 to 0.67) and FDI not severe (0.68 or above) [8, 19].

### Data collection

Initially, the adolescents registered on the electronic database of the Primary Health Care Units in the community were randomly selected. All adolescents and head of families received written information concerning the study aims and procedures. After obtaining written agreement of their participation in the study, data were collected through interviews with adolescents and heads of family, adolescent’s Child-OIDP self-administered questionnaire and clinical oral examination. All interviews were carried out by three dental assistants in a private room at the households to collect information regarding socioeconomic characteristics, FDI and oral health behaviours.

Adolescents were invited until the sample size was achieved. Of those with correct address in the electronic database, 3 did not agree to participate, 2 were excluded due to selection criteria and 7 heads of family did not reply the invitation. The weighted sample comprised 159 participants representing 2004 adolescents.

The study was approved by the Research Ethics Committee of the National School of Public Health, FIOCRUZ (Protocol number 224/09).

### Statistical analysis

Frequency distributions of demographic and socioeconomic families’ status were initially calculated. The association of caries (DMFT = 0, ≥1), Child-OIDP (=0, ≥1) and propensity of adolescents (low, medium and high) with FDI categories (‘not severe’, ‘severe’ and ‘very severe’) was assessed using the Chi-square test. The comparison of mean scores of DMFT and Child-OIDP score among the three groups of FDI was performed by Kruskal–Wallis test. The correlation between the Child-OIDP scores, DMFT and FDI scores was tested using the Spearman Correlation Coefficient.

The comparison of adolescents with oral health needs between normative need and sociodental approach according to FDI groups was tested using the McNemar’s test. The significance level established for all analyses was 5 % (*P* ≤ 0.05). Data were processed using the *Census and Survey Processing System* (CSPro), version 4.0, and the statistical analysis was performed using the *Statistical Package for Social Sciences* (SPSS) version 17.0.

## Results

Demographic and socioeconomic characteristics of the participants are presented in Table 1. Nearly half of the participants were females and the predominant ethnic group among adolescents and heads of families was brown (57.9 and 47.2 %, respectively). Sixty-two percent of heads of families had at least 8 years of schooling. The majority of adolescents were from low-income families (76.7 % with less than 2 minimal wages) and 42.1 % of them were from families receiving financial governmental support. Almost all the houses were built with cement or brick (98.1 %) and 71.1 % of households have access to drinking water (Table 1).

**Table 1:** Demographic and socioeconomic characteristics of adolescents, heads of families and families, Manguinhos, Rio de Janeiro, Brazil, 2010

Variables	%	95 % CI
Adolescents		
Sex		
Female	50.9	48.8–53.1
Male	49.1	46.9–51.3
Ethnicity		
White	31.4	29.4–33.5
Brown	57.9	55.7–60.0
Black	10.7	9.3–12.1
Schooling (years)		
3–5	20.7	18.9–22.4
6	36.1	34.0–38.3
≥7	43.2	41.0–45.4
Head of family		
Sex		
Female	91.2	90.0–92.4
Male	8.8	7.6–10.1
Ethnicity		
White	31.4	29.4–33.5
Brown	47.2	45.0–49.4
Black	20.1	18.4–21.9
Yellow	1.3	0.7–1.8
Kinship		
Father or mother	79.9	78.1–81.6
Uncle	8.2	7.0–9.4
Grandmother or grandfather	8.2	7.0–9.4
Brother or sister	3.7	2.9–4.6
Schooling (years)		
≤4	20.1	18.4–21.9
5–8	42.2	40.0–44.3
≥9	37.7	35.6–39.9
Family		
Family income		
≤1 MW*	37.7	35.6–39.9
>1–2 MW	39.0	36.9–41.1
>2 MW	23.3	21.4–25.1
Housing conditions Material used in the construction of the house		
Cement or brick	98.1	97.5–98.7
Wood	1.9	1.3–2.5
Access to drinking water		
Yes	71.7	69.7–73.7
No	28.3	26.3–30.3
Financial governmental support		
Yes	42.1	40.0–44.3
No	57.9	55.7–60.0

* 1 MW ≈ 315US$

The mean of DMFT was 1.56 (SD = 1.89), ranging from 0 to 10 (median = 1.0); 40.9 % of the adolescents had no dental caries experience (DMFT = 0). Nearly half of the sample reported 0–3 times daily frequency of intake of food/drink with sugar (47 %). Intake of food/drink with sugar 4–5 times daily and 6 or more were 18.9 % and 34 %, respectively. Most of participants reported two or more daily tooth brushing (84.3 %), 12.6 % reported tooth brushing once a day and 3.1 % reported not tooth brushing every day. All adolescents reported regular use of fluoridated toothpaste. Dental attendance was poor. The frequency participants who reported pattern of dental attendance as ‘always’, ‘sometimes’ and ‘rarely’ was 8.2, 22.0 and 69.8 %, respectively. The prevalence of at least one impact of oral health on daily activities (Generic Child-OIDP ≥1) in the last three months was 76.1 %. The impact on ‘eating’ (52.8 %), ‘mouth hygiene’ (37.1 %), ‘smiling’ (37.1 %) and ‘emotional state’ (31.4 %) were the most prevalent. The score of Generic Child-OIDP ranged from 0 to 53, mean 9.66 (SD = 11.3) and median of 5.56. Regarding the intensity of impact, 13.2 and 14.5 % of the sample reported severe and very severe impacts. When CS-Child-OIDP was applied, 64.8 % of adolescents had impact of oral health on daily activities.

The score of CS-Child-OIDP ranged from 0 to 52.78, mean 10.94 (SD = 11.77) and median of 10.95. Oral health-related quality of life scores (Generic Child-OIDP) were positively associated with dental caries experience (DMFT) (rs = 0.163, *P* < 0.05), and inversely associated with FDI scores (rs = −0.174, *P* < 0.05). Dental caries (DMFT) was also negatively correlated with FDI scores (rs = −0.188, *P* < 0.05). When CS-Child-OIDP was applied, oral health-related quality of life scores were also positively associated with dental caries experience (DMFT) (rs = 0.176, *P* < 0.05), and inversely associated with FDI scores (rs = −0.207, *P* < 0.05).

Statistical significant differences were found for the three groups of FDI with respect the occurrence of at least one impact on daily activities (Generic and CS-Child-OIDP ≥1), at least one dental caries and propensity to adopt oral health-related behaviours (*P* < 0.05) (Table 2). The ‘very severe’ FDI group had greater proportions of adolescents with at least one impact on daily activities (79.0 and 66.7 % when Generic and CS-Child-OIDP was applied, respectively), at least one DMFT (66.7 %) and low propensity of oral health-related behaviours (53.1 %). On the other hand, the lower frequencies of these measures were found in the ‘not severe’ FDI group. The mean DMFT and Generic Child-OIDP were statistically higher in the ‘very severe’ and ‘severe’ FDI groups compared to ‘not severe’ FDI group (Table 2). The differences of the mean DMFT and Generic or CS-Child-OIDP were statistically significant between all groups of FDI (*P* < 0.01), except for DMFT between FDI ‘severe’ and ‘not severe’ (*P* = 0.052).

**Table 2:** Oral health-related quality of life (OHRQoL), dental caries (DMFT index) and levels of propensity of adolescents by the family development index (FDI) groups considered, Manguinhos, Rio de Janeiro, Brazil, 2010

	FDI not severe	FDI severe	FDI very severe	*P* value
OHRQoL (generic child-OIDP)				
Mean (SD)	4.28 (4.75)	8.06 (9.23)	11.76 (12.86)	0.001*
≥1	66.9	74.3	79.0	≤0.001**
OHRQoL (specific child-OIDP)				
Mean (SD)	3.50 (3.21)	3.11 (2.80)	3.15 (2.81)	0.001*
≥1	50.0	65.1	66.7	≤0.001**
Dental caries (DMFT)				
Mean (SD)	1.00 (1.70)	1.39 (1.69)	1.78 (2.03)	≤0.001*
≥1	33.1	54.6	66.7	≤0.001**
% ‘D’ of DMFT	41.7	59.8	82.6	≤0.001**
% Severe caries	0.6	5.0	13.8	≤0.001**
Propensity				≤0.001**
Low	33.1	37.9	53.1	
Medium	25.2	28.8	24.7	
High	41.7	33.3	22.2	

* *P* value refers to Kruskal–Wallis

** Chi-square test

**Fig. 2:**
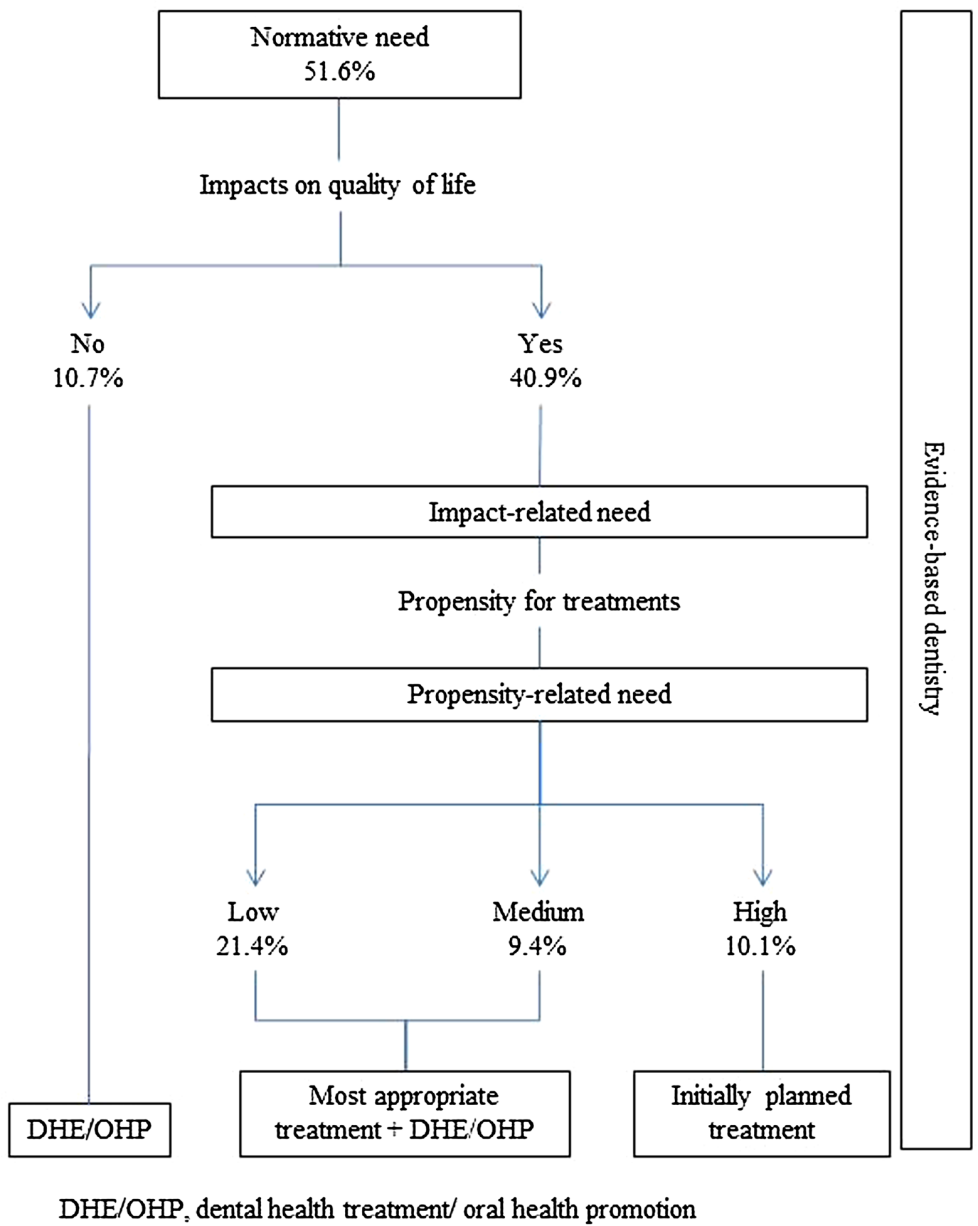
Oral health needs according to normative method and the sociodental approach of 12-year-old adolescents. *DHE*/*OHP* dental health education and oral health promotion

The overall proportion of adolescents with normative need for dental caries was 51.6 %, of which 40.9 % had oral health impact on daily activities (CS-OIDP ≥1). Of these adolescents, 10.1 % were classified as high level of propensity and would be suitable for immediate dental treatment. In addition to clinical intervention, 30.8 % should receive oral health promotion activities (Fig. 2). The proportion of adolescents with normative need was statistically higher than adolescents with impact related need (*P* < 0.001).

**Fig. 3:**
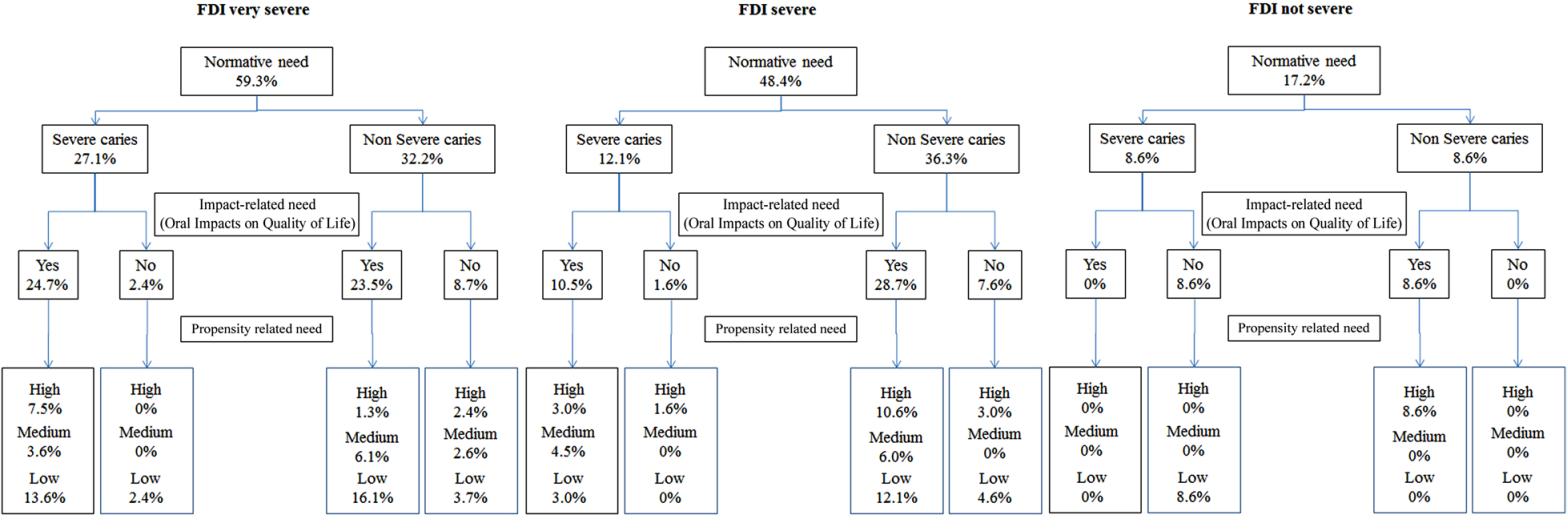
Oral health needs of 12-year-old adolescents using normative method (caries severity), impact-related need and propensity related need according to family living conditions (family development index) groups. *FDI* family development index

The proportion of adolescents with normative need for dental caries in the FDI groups was 59.3 % (FDI ‘very severe’), 48.4 % (FDI ‘severe’) and 17.2 % (FDI ‘not severe’) and was statistically different between FDI groups (*P* < 0.01) (Fig. 3). 27.1, 12.1 and 8.6 % of adolescents with FDI ‘very severe’, ‘severe’ and ‘not severe’ groups were classified with ‘severe caries’ while 32.2, 36.3 and 8.6 % with ‘non severe caries’, respectively. The impact-related needs in these groups were 48.2, 39.2 and 8.6 %, respectively (Fig. 3).

There were significant reductions in normative need for dental caries using the sociodental approach in all FDI groups. Treatment need for dental caries reduced by 50.5 % (from 59.3 to 8.8 %) in the FDI ‘very severe’ group (*P* < 0.01), 34.8 % (from 48.4 to 13.6 %) in the FDI ‘severe’ group (*P* < 0.01), and 8.6 % (from 17.2 to 8.6 %) in the FDI ‘not severe’ group (*P* < 0.01). These reductions were also statistically significant between FDI groups (*P* < 0.05). Only 8.8, 13.6 and 8.6 % of adolescents from FDI ‘very severe’, ‘severe’ and ‘not severe’ groups have impact-related needs and were considered at high level of propensity and, therefore, could start treatment immediately in the respective groups (Fig. 3). Of them, those from very severe FDI and with severe caries should be prioritized to receive dental treatment.

## Discussion

This study compared dental caries treatment needs between normative and the sociodental approach methods in 12-year-old adolescents from families with different living conditions in a deprived community in Brazil. The use of the sociodental approach and information on family living conditions provided relevant information for the organization of oral health services for adolescents. In order to ensure the representativeness of the population of 2004 12-year-old adolescents, a representative weighted sample was randomly selected. Therefore, data from 159 adolescents were expanded using probability weights so that all estimated parameters were representative of the whole population of the 12-year-old adolescents in the investigated community. According to our findings, these measures were a useful tool to distinguish the different levels of dental care needs among adolescents living in a large deprived community in Brazil.

Organization of the resources and provision of oral health services are the core aims of the oral health care systems that are influenced by society structure and cross-cutting societal policies [20]. Healthcare managers and providers’ decisions must ensure that right resources are allocated for the right purpose (allocative efficiency) and the resources allocated to health services will produce the greatest benefit at minimal cost (technical efficiency) [21]. Therefore, adequate information is essential for planning and prioritizing oral health care in order to improve the quality of life and oral health conditions of the population.

Organization of oral health care should be planned based on dental care needs. The information most commonly used in the organization of oral health care is population dental caries experience and prevalence of oral health problems. However, they are unrealistic for planning oral health services because they do not consider constrains and the availability of resources in health care. Dental need evaluation should incorporate not only clinical assessment, but also psychological and social dimensions because the presence of clinical impairment alone is neither necessary nor sufficient basis for need [3]. Based on this concept, the sociodental approach was developed and tested as a new and promising model to assess dental needs overcoming the shortcomings of normative needs. It has been suggested that sociodental approach can assist in organizing the provision of dental care, as it directs the type of health care best suited to individuals so that they can fully benefit from treatment, and that there is a reduction of unnecessary costs in health services [3, 4]. However, sociodental approach is the assessment of dental needs at individual level and it does not incorporate the social structure were individuals are embedded.

Oral health care integrated in primary health care is recognized as a potential model to reduce inequalities in the access and utilization of dental care. In addition, the efficiency of community-based dental services within the primary health context improves when oral health care planning takes into account the socioeconomic characteristics of the individuals, families and neighborhoods [22]. Ignoring the socioeconomic contextual characteristics, such as living conditions of the family, when planning oral health care for children and adolescents is a serious limitation, especially in deprived communities due to the limited resources and barriers for the utilization of dental health services.

The use of the FDI, a measure of living conditions of the family [18, 19], showed a very good capacity to discriminate adolescents’ oral health needs using both normative needs and oral health-related quality of life measurements. The worse the living conditions of the family the higher adolescents’ normative and sociodental needs. A relevant aspect was the capacity of the FDI to characterize the oral health needs of adolescents according to living conditions of the family. Despite living in the same deprived area, oral health needs of the adolescents were ranked according to family socioeconomic background and a clear trend was observed between FDI groups and adolescents’ dental needs considering normative needs and sociodental approach. There are potential benefits in oral health planning when using the sociodental approach combined with FDI compared to normative method. The former has better capacity to define dental treatment priorities and to allocate more efficiently the resources in oral health care. First, FDI is a relevant and low cost tool for screening adolescents from vulnerable families within deprived communities identifying those who are potentially most in need of dental treatment. Second, prioritizing adolescents with severe caries and those with impact-related need will improve functioning and well-being as well as enhancing social interaction and reducing school absence.

Resources for dental care should not be equally distributed for the whole community since the need for dental care among adolescents from poor families (very severe FDI families) was higher than those from better-off socioeconomic families. According to the sociodental approach, individuals with dental needs should start dental treatment while those with moderate and low propensity needs would require oral health education before initiating dental treatment [3, 4, 6]. However, the evidence of the benefit of dental health education in changing health-related behaviours is questionable [23]. Thus, a critical issue when using propensity-related need as part of the sociodental approach is the potential risk to increase inequalities in access to oral health care. The purpose of using propensity-related need is not to determine who should receive treatment or not since treatment must be prescribed to all individuals with progressive oral diseases such as dental caries, even without the impact being assessed [4, 6]. Instead, it allows the identification of priorities among those with different levels of propensity-related need. Adolescents with moderate and low propensity-related need should receive dental treatment and oral health promotion activities.

The use of DMFT as the only clinical measure in combination with Child-OIDP in the impact-related need assessment is another limitation of this study. To minimize this limitation, caries CS-OIDP was applied. However, similarly to a previous study [17], the association between normative treatment need for dental caries and prevalence of oral impacts was statistically significant when both Generic or CS-Child-OIDP were used. The results of both indexes were very similar in our study demonstrating that the impact on adolescent’s daily activities can be assessed either by Generic or caries Specific Child-OIDP when only normative treatment for dental caries is considered. Tooth loss and malocclusion are also oral conditions related to normative need and they can also impact on adolescent’s daily lives. These oral conditions must be considered in future studies.

Similar to previous research, dental need using the sociodental approach was lower compared to the normative method [4, 5, 24, 25]. The evidence is supported by studies involving progressive oral conditions (e.g. dental caries) [5] and malocclusion in adolescents [4], and prosthodontic and periodontal treatment needs assessment in adults [24, 25]. In our study, there was a reduction from 51.6 to 10.1 % on dental caries treatment need when normative need was compared to the sociodental approach. This finding is similar to a previous study of progressive oral conditions in adolescents since the treatment need reduced from 54.4 % (normative need) to 16.6 % (sociodental approach) [5]. Greater discrepancies between normative need and sociodental approach were reported for other oral conditions. The estimate need of orthodontic treatment using the sociodental approach decreased by 70 % compared with normative need [4]. In adults the need of periodontal treatment and prosthodontic treatment using the sociodental approach were 90 and 75 %, respectively, lower than using the normative need method [24, 25].

Despite the relevant implications from the current findings, this study has some limitations. The application of our findings to non-deprived communities and other age groups must be cautious. Further studies involving other population groups, such as children, adults and elderly, are needed.

## Conclusion

Considering the above mentioned limitations of the present study, our findings suggest that using the sociodental approach and FDI on the identification of adolescents’ dental needs can improve the organization of oral health care systems in deprived communities because of the following reasons. First, oral health promotion and dental treatment can be offered focusing on the reduction of inequalities in the utilization of health care as well as directed to those who will benefit more. Second, the offer of dental care takes into account social and subjective aspects related to oral health and not only normative methods. Finally, our findings suggest that a comprehensive approach using individual clinical and subjective measures of oral health as well as the socioeconomic conditions of the family is a helpful tool for organizing oral health care and defining priorities.

## Abbreviations

FDI: Family development index
OHRQoL: Oral health-related quality of life
Child-OIDP: Child oral impacts on daily performance
CS-Child-OIDP: Specific child-OIDP
DMFT Index: Decayed, missing, and filled teeth index
SUS: Sistema Unico de Saude
HDI: Human development index
FIOCRUZ: Fundação Oswaldo Cruz
CSPro: Census and survey processing system
SPSS: Statistical package for social sciences
